# Host species differences in the thermal mismatch of host–parasitoid interactions

**DOI:** 10.1242/jeb.245702

**Published:** 2023-06-27

**Authors:** Katherine H. Malinski, Clyde E. Sorenson, M. Elizabeth Moore, Christopher S. Willett, Joel G. Kingsolver

**Affiliations:** ^1^ Department of Biology, University of North Carolina, Chapel Hill, NC 27514, USA; ^2^ Department of Entomology and Plant Pathology, North Carolina State University, Raleigh, NC 27695, USA; ^3^ Department of Applied Ecology, North Carolina State University, Raleigh, NC 27695, USA

**Keywords:** Climate change, Developmental recovery, *Manduca*, *Cotesia*, Heat stress

## Abstract

Extreme high temperatures associated with climate change can affect species directly, and indirectly through temperature-mediated species interactions. In most host–parasitoid systems, parasitization inevitably kills the host, but differences in heat tolerance between host and parasitoid, and between different hosts, may alter their interactions. Here, we explored the effects of extreme high temperatures on the ecological outcomes – including, in some rare cases, escape from the developmental disruption of parasitism – of the parasitoid wasp, *Cotesia congregata*, and two co-occurring congeneric larval hosts, *Manduca sexta* and *M. quinquemaculata.* Both host species had higher thermal tolerance than *C. congregata*, resulting in a thermal mismatch characterized by parasitoid (but not host) mortality under extreme high temperatures. Despite parasitoid death at high temperatures, hosts typically remain developmentally disrupted from parasitism. However, high temperatures resulted in a partial developmental recovery from parasitism (reaching the wandering stage at the end of host larval development) in some host individuals, with a significantly higher frequency of this partial developmental recovery in *M. quinquemaculata* than in *M. sexta.* Hosts species also differed in their growth and development in the absence of parasitoids, with *M. quinquemaculata* developing faster and larger at high temperatures relative to *M. sexta.* Our results demonstrate that co-occurring congeneric species, despite shared environments and phylogenetic histories, can vary in their responses to temperature, parasitism and their interaction, resulting in altered ecological outcomes.

## Teaser

**Editor's choice:** High temperatures reveal differences between two host species in their ability to recover at least parts of their developmental pathways that are typically disrupted by parasitism.

## INTRODUCTION

As climate change continues, extreme high temperatures and heat wave events are expected to become more common and intense (Portner et al., 2022). How organisms, communities and ecosystems will fare is a critical question in biology (Leadley et al., 2010; Malhi et al., 2020; Parmesan, 2006). Predicting these responses hinges on understanding both the direct effects of temperature on organisms (Hamblin et al., 2018; Dale and Frank, 2014; Huey et al., 2009), as well as the indirect effects on interactions between species (Dale and Frank, 2018; Padfield et al., 2020; Youngsteadt et al., 2019). Species interactions, like all biological processes, are temperature-mediated, and can change in magnitude and direction under different thermal conditions (Comeault and Matute, 2021; Colwell et al., 2012).

Under extreme high temperatures, differing thermal sensitivities among interacting species can affect the ecological outcomes of the interaction, especially for highly dependent species relationships such as hosts and their parasites, pathogens or endosymbionts (Kiers et al., 2010; Cizauskas et al., 2017; Vasseur et al., 2014; Linder et al., 2008). Differences in thermal limits or optima can result in thermal mismatch, in which temperature differentially impacts performance of the interacting species, often resulting in a breakdown of the typical ecological dynamics and outcomes of the interaction (Rohr and Cohen, 2020; Sauer et al., 2019). This phenomenon has been observed in a variety of interacting species, including coral and zooxanthellae (Herrera et al., 2021; Hoegh-Guldberg, 1999), insect hosts and parasitoids (Agosta et al., 2018; Moore et al., 2021; Seehausen et al., 2017), amphibians and fungi (Cohen et al., 2017; Sauer et al., 2018; 2019), insects and microbiota (Wernegreen, 2012), marine invertebrates and parasites (Gehman et al., 2018) and others.

Although thermal sensitivity is often phylogenetically conserved (Huey and Bennett, 1987; Angilletta, 2009), closely related species can also differ in their response to temperature. For example, congeneric species that occur at different latitudes, elevations or habitats may exhibit differences in thermal tolerance, thermal optima or temperature limits for growth and development (Ravaux et al., 2016; Leong et al., 2018). Less is known about thermal differences between sympatric congeners that co-occur in similar habitats, seasons and geographic areas. Such species are often subjected to shared natural enemies such as parasites and pathogens, which can often infect more than one permissive host (Rutrecht and Brown, 2009). This raises the possibility that differences in thermal sensitivities among hosts can affect the ecology of host–parasite interactions for parasites that utilize multiple co-occurring hosts. The degree to which complex, multi-species host–parasite systems will respond to climate change may depend on differing host performance driven by parasitism, as well as differences in host and parasite performance driven by temperature (Gehman et al., 2018; Schampera et al., 2022). However, the magnitude and/or direction of such shifts may differ by host species. Here, we explore this possibility by investigating the effects of extreme high temperature events on the interactions between a parasitoid and two common co-occurring insect hosts.

*Cotesia congregata* (Say 1836) (Hymenoptera: Braconidae) is a gregarious endoparasitoid wasp that develops inside the body of a larval lepidopteran host during its egg and larval stages (Beckage and Riddiford, 1983; Adamo et al., 2016; Beckage et al., 1994). Like other braconid parasitoids, *C. congregata* harbors an endosymbiotic polydnavirus, the *C. congregata* bracovirus (CcBV), which is essential to the success of parasitism and wasp reproduction (Dushay and Beckage, 1993; Adamo et al., 2016; Strand and Burke, 2014). In a parasitism event, adult female *C. congregata* oviposit eggs, venom and CcBV virions into the hemocoel of the larval host. The CcBV incorporates into the host genome, where it expresses viral genes, disrupting immune and endocrine pathways in the host (Dushay and Beckage, 1993; Chevignon et al., 2015; Strand and Burke, 2014). During the first ∼5 days post-oviposition, the CcBV immunosuppresses the host, protecting wasp eggs from host immune defenses, including the encapsulation and melanization pathways important to parasite and parasitoid defense (Chevignon et al., 2015; 2014; Adamo et al., 2016). After ∼5 days post-oviposition, wasp eggs hatch, hosts regain immune function and wasp larvae evade host immunity via other mechanisms (Adamo et al., 2016). During the later larval stages of *C. congregata* development, the CcBV manipulates the host's endocrine system and halts host development in the final instar to prevent host pupation; wasp larvae then chew through the host cuticle, spin cocoons and remain attached to hosts during pupation before eclosing as adults (Beckage and Templeton, 1986; Beckage and Riddiford, 1983). Parasitized hosts can survive as terminal instar larvae for days or weeks after wasp emergence, but remain developmentally disrupted by the CcBV, and do not typically progress into the prepupal wandering stage, pupation or adulthood. As with most parasitoids, parasitization by *C. congregata* inevitably leads to host death prior to host reproduction (Dushay and Beckage, 1993).

In the southeastern USA, *C. congregata* is an effective biocontrol against several sphingid agricultural pests, including larvae of the tobacco and tomato hornworms, *Manduca sexta* (Linnaeus 1763) (Lepidoptera: Sphingidae) and *M. quinquemaculata* (Haworth 1803) (Lepidoptera: Sphingidae), respectively (Kester et al., 2002). Parasitism exerts a strong selective pressure on both host species*,* resulting in up to 90% of observed larval host mortality in the field (Lawson, 1959). Although *M. sexta* is a model system in insect physiology, development and immunology (Thorpe and Barbosa, 1986; Beckage et al., 1990; Nijhout, 1976; Reynolds and Nottingham, 1985; Adamo and McMillan, 2019; Adamo et al., 2010), little is known about the thermal biology of *M. quinquemaculata* owing to their difficulty in laboratory rearing. Both host species feed on solanaceous plants, and co-occur spatially and temporally across North America, often on the same host plant during the same life stage, though *M. quinquemaculata* is more common in the northeastern portion of this range, and *M. sexta* in the southeastern Gulf Coast states (Capinera, 2008; Lotts and Naberhaus, 2017; Hodges, 1971; Kester et al., 2002). Both host species suffer similar rates of parasitism by *C. congregata* in the American Southeast (Lawson, 1959) (K. Malinski and C. Sorenson, personal observations).

Differences in thermal limits among interacting hosts, parasites and symbionts are not uncommon in nature, and the effects of extreme high temperatures on complex ecological systems can be disruptive (Agosta et al., 2018; Seehausen et al., 2017; Palmer-Young et al., 2018; Roberts et al., 2018; Malhi et al., 2020). In this focal system, *Manduca* larvae are thermal generalists that can feed, grow and develop over a wide range of temperatures (Reynolds and Nottingham, 1985; Kingsolver et al., 2015). Previous studies using *M. sexta* from a laboratory colony demonstrated that *C. congregata* has a lower thermal tolerance than its *M. sexta* host – high temperatures that are non-stressful to hosts result in complete mortality of developing parasitoids (Moore et al., 2020, 2021). Although high temperatures kill developing *C. congregata* eggs in *M. sexta* hosts, the parasitoid's viral endosymbiont CcBV remains at least partially functional, disrupting host development in the final larval instar and preventing the prepupal and pupal stages even in the absence of emerging parasitoids. These hosts, called WOWEs (WithOut Wasp Emergence; Moore et al., 2020) continue to eat throughout the final larval instar, grow to enormous sizes (e.g. ≥12 g, up to as large as ∼19 g; Moore et al., 2021) and eventually die as abnormally long-lived larvae (Moore et al., 2020); similar phenotypes are achieved by injecting hosts with purified CcBV lacking wasp eggs (Beckage and Gelman, 2001). High temperatures commonly result in decreased parasite or pathogen load in many systems across different taxa (Sauer et al., 2019; Seehausen et al., 2017; Agosta et al., 2018), but it is presently unclear whether high temperatures can lead to a recovery of CcBV-disrupted developmental pathways in parasitized hosts in this typically lethal host–parasitoid–endosymbiotic virus interaction.

Recent and preliminary studies suggest host evolutionary history may play an important role in determining whether high temperatures can facilitate partial developmental recovery from parasitism. In *M. sexta* hosts from a domesticated laboratory colony, ∼10% of parasitized hosts display normal (i.e. resembling unparasitized) development into prepupal wanderers under both control and high temperature conditions, and were assumed to result from failed parasitoid ovipositions (Moore et al., 2021). However, in a preliminary study using *M. sexta* hosts from wild field-collected populations in North Carolina, parasitized host development into prepupal wanderers was observed in a significantly higher proportion of hosts under high temperature conditions relative to control temperature conditions. Failed ovipositions were not ruled out as a cause (i.e. host tissues were not tested for presence of CcBV), but the temperature-dependent results of these studies suggest host population-level differences may affect the hosts' abilities to recover the CcBV-disrupted development of the prepupal wandering stage under high temperatures. Laboratory and field-sourced *M. sexta* differ physiologically, with laboratory *M. sexta* displaying decreased immunity and heat tolerance relative to their field counterparts – key pathways in the response to parasitism and temperature (Alston et al., 2020; Kingsolver and Nagle, 2007; Diamond and Kingsolver, 2010). How these host differences shape the ecological outcomes of this host–parasitoid interaction, and how the ecologically and agriculturally relevant congeneric host *M. quinquemaculata* is affected by high temperatures under parasitism, is unknown. Investigating these population- and species-level differences may provide insights into the mechanisms underlying thermal mismatch in this and other host–parasitoid systems.

In the southeastern US, both *M. sexta* and *M. quinquemaculata* occupy similar ecological niches, including habitat, thermal environment, selective pressures imposed by the parasitoid (and other parasites and diseases) and host plants, and share a close phylogenetic relationship (Lawson, 1959; Hodges, 1971; Kawahara et al., 2013). *Cotesia congregata* parasitizes both *Manduca* hosts with no known difference in success between the two species in the field (Lawson, 1959) (K. Malinski and C. Sorenson, personal observations), but congeneric species can differ in their response to temperature, parasitism and their interaction. We determined: (1) whether *C. congregata* also displays a thermal mismatch in the parasitism of *M. quinquemaculata* hosts, (2) whether parasitized hosts of each species can partially recover CcBV-disrupted development under high temperatures, and (3) whether sympatric congeners differ in the magnitude and direction of their response to temperature and parasitism.

## MATERIALS AND METHODS

### Experimental animals

*Manduca* hosts were collected as eggs from the undersides of tobacco leaves at Central Crops Research Station (Clayton, NC, USA) and Upper Coastal Plains Research Station (Rocky Mount, NC, USA) in the summer (June–September) of 2021 and brought to a laboratory environment at the University of North Carolina-Chapel Hill (Chapel Hill, NC, USA). Owing to lower abundance in the field, *M. quinquemaculata* host numbers were supplemented by light-trapping adult females, placing them in tents in the laboratory with tobacco plants for several days to collect eggs. All eggs were placed on tobacco leaves (strain NC919) and kept at 25°C under a 14 h:10 h light:dark photoperiod in environmental chambers (Percival Scientific 36VL) until 3rd instar (mortality prior to this point was ignored). Larval hosts were fed tobacco leaves *ad libitum* throughout rearing and for the duration of the experiment. A 14 h:10 h light:dark photoperiod was used for the entirety of the experiment.

*Cotesia congregata* (Say 1836) wasps were sourced from a laboratory colony at UNC-Chapel Hill; the colony is maintained at room temperature (25°C) under a 14 h:10 h light:dark photoperiod, fed a 60% honey–agar solution, and given water via moist sponges. Fourth instar *M. sexta* hosts from a laboratory colony at UNC-Chapel Hill are parasitized by adult females in the colony to rear new wasps and perpetuate generations. The colony was founded in 2017 with wasps sourced from the Virginia Commonwealth University colony (Richmond, VA, USA) that originated from field collections in southeastern VA; the UNC colony is supplemented annually with wasps from the aforementioned field sites in Clayton and Rocky Mount, NC, USA (Moore et al., 2020).

### Experimental design

This study consisted of two treatment types (parasitism, temperature) and two levels per treatment: parasitized (P) or non-parasitized control (NP), and heat shock (40°C) or control (25°C) temperatures during a 24-h period starting on the day of parasitism. The length and temperature of the heat shock was chosen based on lethality to developing wasps but not *M. sexta* hosts; experimental results of this treatment are similar to those produced by a more ecologically relevant 3-day heat wave with a 30±10°C diurnal thermocycle (Moore et al., 2021), but 24 h at constant temperatures was used for ease of experimental manipulation and control of the critical period in this study.

The experiment was fully factorial with four treatment groups (P/40°C, P/25°C, NP/40°C, NP/25°C) per host species (*M. sexta* and *M. quinquemaculata*), resulting in eight experimental groups total. Larger sample sizes were used in the P/40°C treatment groups to better characterize the expected low frequencies of developmental recovery of the wandering stage. In the morning of the first day of 3rd instar, hosts from each species were randomly assigned to one of the four treatment groups, weighed and placed in individual experimental containers with tobacco leaves. Hosts in the parasitized (P) treatment groups were exposed to adult female *C. congregata* until at least one oviposition event lasting >2 s was observed; oviposition events shorter in duration were considered unsuccessful based on previous studies (Moore et al., 2020, 2021, 2022). If more than three oviposition events were observed, the host was excluded from the study. All hosts were placed in their rearing 25°C environmental chamber until mid-afternoon of the same day, then placed in new environmental chambers at their respective treatment temperatures (40°C or 25°C) for 24 h.

Following the 24-h temperature treatment period, hosts were weighed and tobacco leaves were replaced. All hosts were returned to their original 25°C environmental chamber for the remainder of the experiment; tobacco leaves were replaced as needed, and date and mass at each molt were recorded. Hosts exited the experiment when one of the following developmental outcomes was observed: wasp emergence (wasp larvae or cocoons visible outside the host cuticle), pre-pupal wandering (both visible dorsal vessel and wandering behavior observed), WOWE (hosts WithOut Wasp Emergence, defined as survival 3 weeks past the date of the last molt, including extranumery molts, without wandering) or mortality after entry into the experiment but prior to any of the aforementioned outcomes. The WOWE development cutoff was chosen based on previous studies demonstrating that developmental recovery of the wandering stage in P/40°C hosts occurs within 3 weeks of the last molt (Moore et al., 2021, 2022). Therefore, the mass and age of hosts with the WOWE developmental outcome was taken at this semi-arbitrary 3 week cutoff point, rather than a discreet biological time point like the other developmental outcomes.

To determine whether development into the prepupal wandering stage in parasitized hosts was due to failed oviposition, polymerase chain reaction (PCR) was performed to confirm successful parasitism events in all wanderers of the parasitized groups. Primers specific to the CcBV gene *cystatin-1* were modified from Serbielle et al. (2008): forward 5′-GTAAGGACAGTTTTTATCTAG-3′ and reverse 5′-ATGGGCAAGGAATATCGAGTG-3′. Upon observation of wandering (defined above), hosts were bled from the dorsal horn and DNA was extracted from ∼200 μl of collected hemolymph (DNeasy Blood & Tissue Kit, Qiagen) using a modified insect tissue-specific protocol (Coffman et al., 2020). PCR conditions were: initial denaturation at 94°C for 2 min followed by 40 rounds of denaturation at 94°C for 30 s, annealing at 50°C for 1 min, extension at 72°C for 1 min 30 s, and a final extension at 72°C for 3 min. DNA extracted from the hemolymph of a parasitized *M. sexta* larva with prior wasp emergence served as a positive control, and that from an unparasitized larva as a negative control. Two technical replicates were run for each biological sample; samples without presence of a band in the *cystatin-1* reaction for both replicates were assumed to be failed parasitism events and were excluded from analyses. Parasitized wanderers that tested positive for *cystatin-1* were placed in pupation boxes and monitored for death or pupation and subsequent eclosion.

### Statistical analyses

#### Survival analyses

Cox proportional hazards (coxph) models were used to evaluate mortality prior to other developmental outcomes across species and treatment groups starting at 3rd instar. Models were fit to a Kaplan–Meier curve using the ‘*coxph*’ and ‘*Surv*’ functions in the *survival* package (v 3.2.13) in R (v 4.1.2) with temperature, species and parasitism status as fixed variables. Model fits including the sum of fixed variables, as well as their interaction, were compared with each other and a null (intercept-only) model via Akaike’s information criterion (AIC) and analyses of deviance (ANODEVs). Mortality was defined as death prior to any other developmental outcome (wasp emergence, wandering or WOWE) after entry into the experiment at 3rd instar; these individuals were excluded from subsequent analyses.

#### GLM analyses of wandering stage recovery

To evaluate the effect of temperature on prepupal wandering stage recovery in the two host species, generalized linear models (GLMs) with a binomial distribution and logit link were employed using the ‘*glm***’** function in the *dplyr* package (v 1.0.8) in R. Temperature and species were included as fixed variables and observation of wandering was included as the binary response variable. ANODEVs were run on all model fits. The model including both fixed variables as well as the model including the additional interaction term were indistinguishable via AIC, so both are reported. Because unparasitized *Manduca* develop normally into prepupal wanderers, unparasitized treatment groups were excluded from the GLM and multinomial logistic regression analyses (below).

#### Multinomial analyses of developmental outcomes

To evaluate the effects of temperature and host species on all possible developmental outcomes (wasp emergence, WOWE, wandering), multinomial models were employed using the ‘*multinom***’** function in the *nnet* package (v 7.3.16) in R. The effects of temperature, species and their interaction were assessed by successively dropping the term of interest from the model and comparing each model in a pairwise fashion via ANODEV. The model including both predictor terms as well as the model including the additional interaction between predictors were indistinguishable via AIC. As stated above, only parasitized treatment groups were included in the multinomial analyses.

#### Growth and development

Host species growth and development were compared across all treatment groups. Unparasitized *Manduca* displayed only the wandering outcome; time to and mass at wandering were evaluated in these treatment groups (NP/25°C and NP/40°C) via two-way ANOVAs with species and temperature as interacting predictor terms. In the parasitized, control temperature treatment group (P/25°C), species-specific differences in time to and mass at wasp emergence were evaluated via unpaired *t*-tests. Finally, we performed *post hoc* analyses of extranumery instar development in the double-treatment group (P/40°C). *Manduca* typically wander and molt to pupation after 5 larval instars, but can develop extranumery (6+) instars under stress (Kivelä et al., 2020; Abarca et al., 2020). Species-specific differences in the development of extranumery instars in the P/40°C group were evaluated using Chi-square statistics for all surviving P/40°C hosts, then separately for WOWE and wandering hosts within this treatment group to evaluate outcome-specific effects.

## RESULTS

### Confirming wandering stage recovery

**Fig. 1.**
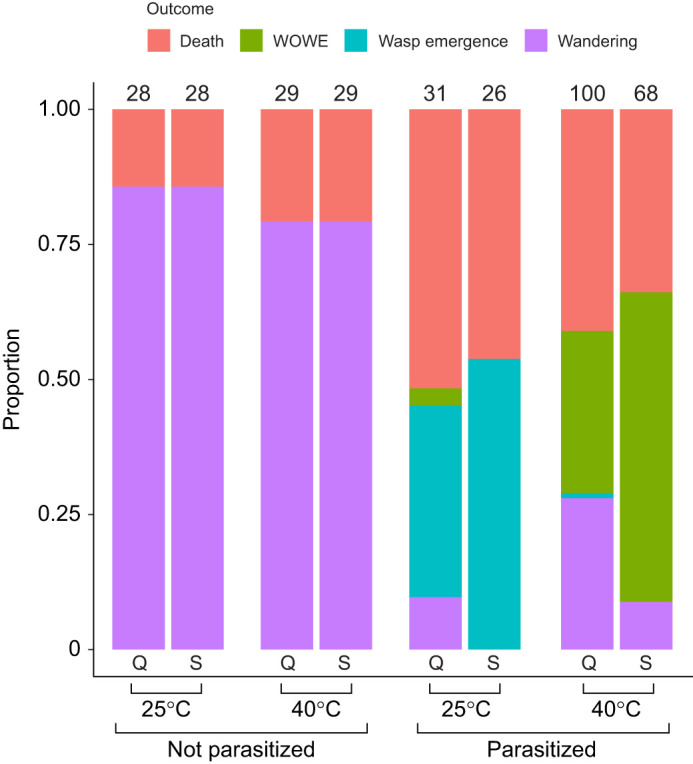
**Proportions of host species developmental outcomes by treatment group.** Outcomes were quantified starting from the onset of 3rd instar by temperature (40°C, 25°C) and parasitism (P, parasitized, NP, not parasitized) treatment groups for *Manduca sexta* (S) and *M. quinquemaculata* (Q) host species*.* Colors indicate host outcomes [pink, death; green, WithOut Wasp Emergence (WOWE); teal, wasp emergence; purple, wandering]. Numbers above bars indicate sample sizes of respective treatment groups for individuals entering into the experiment at 3rd instar.

A primary goal of this study was to determine the extent to which host species recovered development of the wandering stage following parasitism and exposure to high temperatures. Out of 39 parasitized hosts that exhibited wandering, 37 tested positive for presence of CcBV via PCR; the two negative individuals (both *M. quinquemaculata*) were excluded from further analyses. Although both host species exhibited some degree of novel developmental recovery of the wandering stage in the P/40°C groups, the frequency was species-specific (Fig. 1). *Manduca quinquemaculata* recovered the prepupal wandering stage in 47% (*n*=28) of surviving parasitized, heat-shocked hosts, compared with 13% (*n*=6) for *M. sexta*. No *M. sexta* hosts in this treatment group successfully pupated, compared with 3.3% (*n*=2) of *M. quinquemaculata* hosts. Of these, only one eclosed as an adult, but the wings and proboscis of this individual were malformed.

Partial wasp emergence (two cocoons) was observed in one *M. quinquemaculata* host in the P/40°C group; neither wasp eclosed successfully. No wasps emerged from any *M. sexta* hosts in this treatment group. All *M. sexta* hosts in the P/25°C treatment group displayed wasp emergence. Surprisingly, 9.6% (*n*=3) of *M. quinquemaculata* hosts in the P/25°C treatment group regained the prepupal wandering stage (Fig. 1) and were confirmed to have been successfully parasitized via hemolymph PCR assay; none of these successfully pupated.

### Survival analyses

**Fig. 2.**
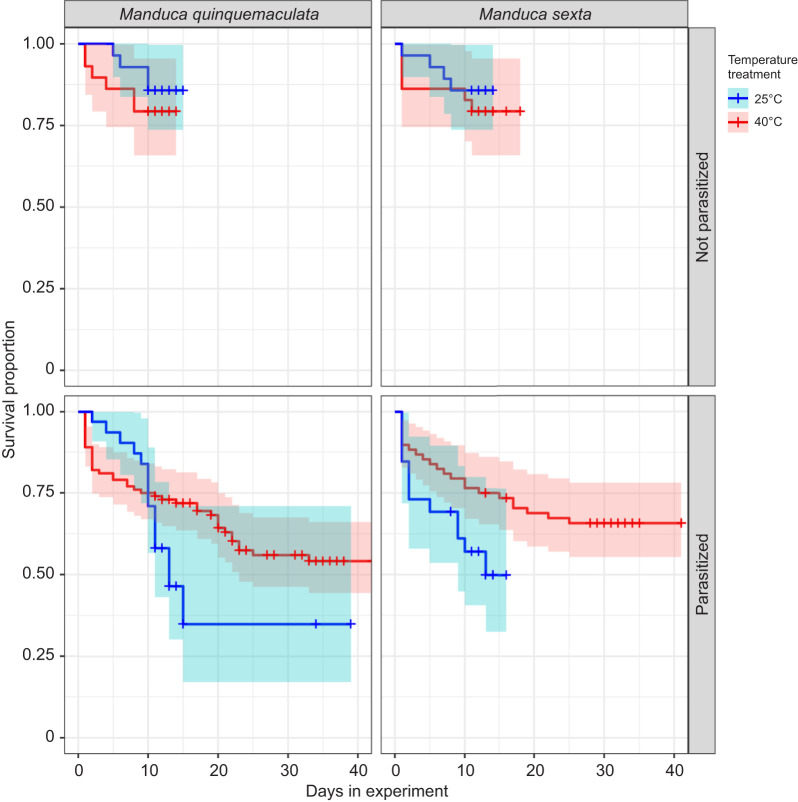
**Kaplan–Meier curves displaying probabilities and confidence intervals of host survival over time from experimental onset at 3rd instar.** The panels are divided by parasitism status (top panels, not parasitized; bottom panels, parasitized) and species (left panels, *M. quinquemaculata*; right panels, *M. sexta*). Temperature treatment is indicated by line color (blue, 25°C; red, 40°C). Plus signs indicate censoring of individuals upon observation of developmental outcomes (wandering, wasp emergence, WOWE). Mortality is defined as death prior to developing another outcome (listed in the previous sentence) after 3rd instar.

We evaluated the effects of temperature and parasitism on mortality of each host species after entry into the experiment at 3rd instar*.* Mortality is defined as death prior to observation of wasp emergence, wandering or development into a WOWE host after entry into the experiment at 3rd instar. The coxph model fit including fixed variables of temperature, species and parasitism (additive model) did not differ significantly from the model including their interaction terms (interactive model) (ANODEV; d.f.=4, *P*=0.26), but had a slightly lower AIC, so the additive model is reported here. The additive model differed significantly from the null model (ANODEV; d.f.=3, *P*=0.01). Probability of survival did not vary by host species (coxph; *P*=0.48), but did vary significantly with parasitism (coxph; *P*<0.01) and marginally with temperature (coxph; *P*=0.071). Model coefficients are reported in Table S1. For both species, parasitization significantly reduced the probability of survival to another developmental outcome (wasp emergence, WOWE or wandering) (Figs 1 & 2).

### GLM analyses of wandering stage recovery

Because unparasitized hosts only display wandering developmental outcomes, both the GLM and multinomial analyses (below) included only parasitized hosts. In the GLM analyses, the models including temperature and species as predictor terms, as well as the model including the additional interaction between predictors, were indistinguishable via AIC. In the full model, temperature and species were both significant predictors of deviance in wandering stage recovery (Chi-square test; d.f.=1, *P*=0.01 and *P*<0.01, respectively), but the interaction term was not significant (Chi-square test; d.f.=1, *P*=0.31). High temperatures increased the frequency of partial developmental recovery from parasitism, with a higher rate of wandering stage recovery in *M. quinquemaculata* than in *M. sexta*.

### Multinomial analyses of developmental outcomes

The multinomial logistic regression analyses evaluated the frequency of each developmental outcome (wasp emergence, WOWE and wandering) across treatment groups and host species. The model that included both temperature and species as fixed variables was indistinguishable from the model including the additional interactive term between variables according to AIC and *P*-value (Table 1). Both of these models outperformed the simpler models, suggesting that both species and temperature, but not their interaction, were significant predictors of developmental outcomes in parasitized *Manduca.*

**Table 1.**
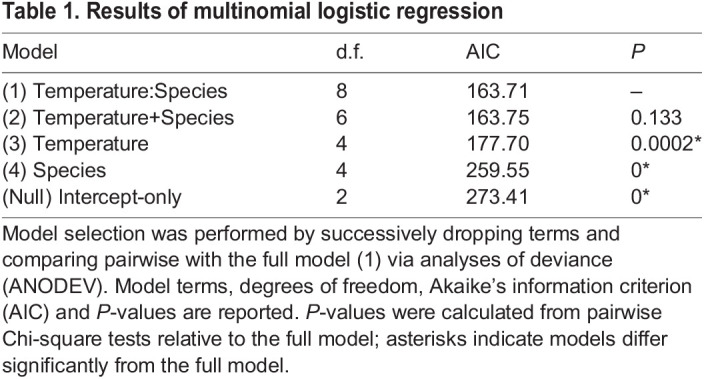
Results of multinomial logistic regression

### Growth and development

**Fig. 3.**
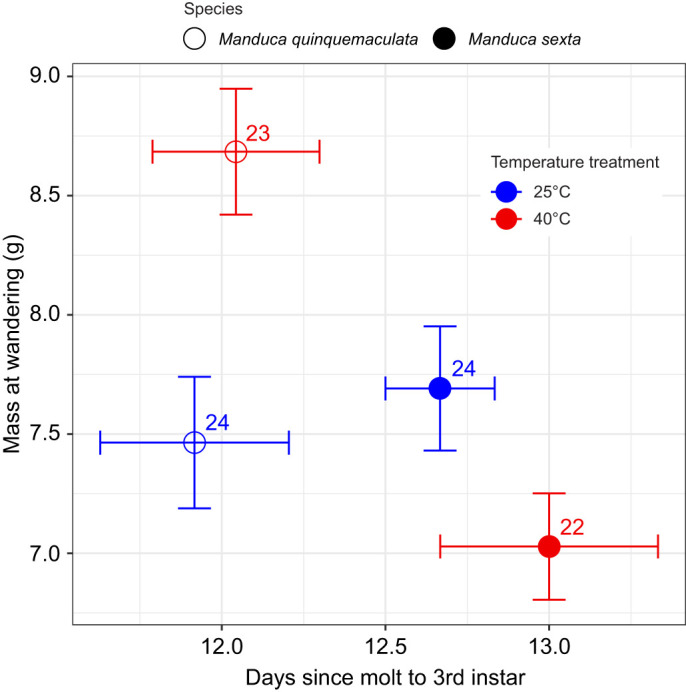
**Unparasitized host larval mass (g) and experimental age (days) at the wandering developmental outcome.** Means and standard errors are shown. Color indicates treatment temperature (blue, 25°C; red, 40°C); symbol fill indicates host species (open circles, *M. quinquemaculata*; filled circles, *M. sexta*). Numbers beside symbols indicate sample sizes. Experimental age began on the first day of molt to 3rd instar and ended on the day wandering was observed.

Growth and development of *Manduca* hosts was evaluated across treatment groups. In the non-parasitized (NP) groups (NP/40°C, NP/25°C), all surviving individuals wandered as expected (Fig. 1). Under both temperature treatments, *M. quinquemaculata* developed significantly faster than *M. sexta* (Fig. 3). Species was a significant predictor for development time of unparasitized hosts in the two-way ANOVA test (ANOVA; d.f.=1, *F*=10.2, *P*<0.01), but temperature and the interaction term were insignificant (temperature ANOVA; d.f.=1, *F*=0.74, *P*=0.39; species×temperature ANOVA; d.f.=1, *F*=0.15, *P*=0.69). For mass at wandering, the interaction between species at temperature was significant: at 25°C, *M. quinquemaculata* were, on average, smaller than *M. sexta*, but the inverse was observed at 40°C (Fig. 3). In the two-way ANOVA test, the interaction between species and temperature (ANOVA; d.f.=1, *F*=13.3, *P*<0.01), and species alone (ANOVA; d.f.=1, *F*=7.08, *P*<0.01) were significant predictors of mass at wandering, and temperature alone was insignificant (ANOVA; d.f.=1, *F*=1.25, *P*=0.26).

**Fig. 4.**
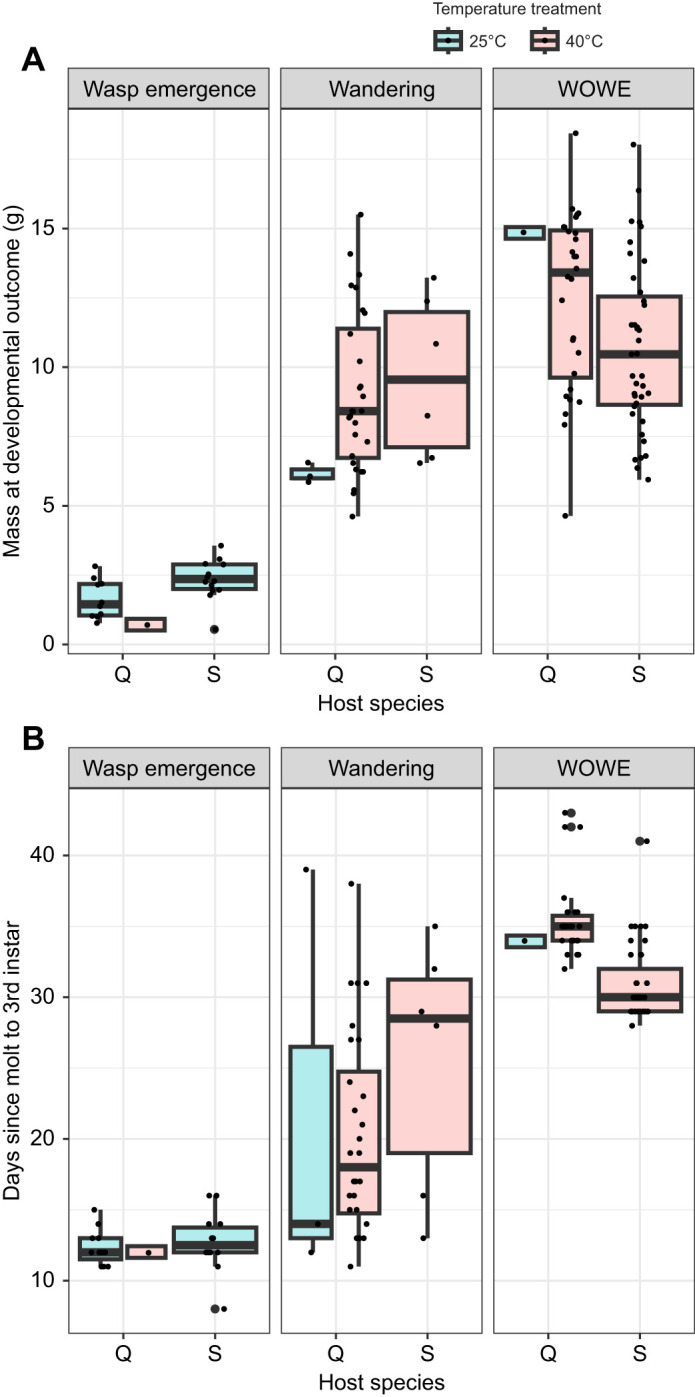
**Boxplots depicting means and standard deviations in age and mass of parasitized hosts at their final developmental outcome timepoint.** (A) Parasitized host larval mass (g) and (B) experimental age (days) at final developmental outcome (wasp emergence, wandering, WOWE). Color indicates treatment temperature (blue, 25°C; red, 40°C). Species is shown on the *x*-axis within each panel (Q, *M. quinquemaculata*; S, *M. sexta*). Because *M. quinquemaculata* from both temperature treatments developed into every developmental outcome, two boxplots are shown for each Q position on the *x*-axis (left, 25°C; right, 40°C). Because *M. sexta* hosts with the wasp emergence outcome exclusively originated from the 25°C group, and hosts with the wandering or WOWE outcome exclusively originated from the 40°C group, only one bar is shown for each S position on the *x*-axis. Experimental age began on the first day of molt to 3rd instar and ended on the day the respective developmental outcome (wasp emergence, wandering, WOWE) was observed.

Surviving hosts of both species in the parasitized, control temperature groups (P/25°C) predominantly displayed the wasp emergence outcome (Fig. 1). Host species with this outcome differed significantly in their mass at (Fig. 4A), but not time to (Fig. 4B), emergence of parasitoids. On average, *M. quinquemaculata* were significantly smaller than *M. sexta* at the observed emergence of wasps (*t*-test; *t*=–2.79, d.f.=21.5, *P*=0.01), and developed slightly faster, but this difference was insignificant (*t*-test; *t*=–0.59, d.f.=21.8, *P*=0.56).

Extranumery instars were observed in the P/40°C group for both species, and in the P/25°C group for one *M. quinquemaculata* individual (this individual was one of two in this treatment group that developed the WOWE outcome). In the P/40°C group, 69.3% (*n*=52) of *M. quinquemaculata* molted to extranumery instars, compared with 29.6% (*n*=16) of *M. sexta*; this species-specific difference was significant (Chi-square test; d.f.=1, *P*<0.01). To determine whether developmental outcome type was correlated with this phenomenon, wanderers and WOWEs in the P/40°C group were subsequently analyzed separately. Of the individuals that recovered the prepupal wandering stage, 38.7% (*n*=12) of *M. quinquemaculata* developed extranumery instars, compared with 50% (*n*=3) of *M. sexta*; the sample size of *M. sexta* wanderers in this treatment group was too low to determine significant differences between host species. Of the individuals that developed into the WOWE outcome, significantly more *M. quinquemaculata* hosts developed extranumery instars (96.7%, *n*=30), compared with 25.6% (*n*=20) of *M. sexta* hosts (Chi-square test; d.f.=1, *P*<0.01). The frequency of hosts developing extranumery instars for each outcome is shown in Figs S1 and S2.

## DISCUSSION

We sought to determine (1) whether parasitized *M. quinquemaculata* (like its co-occurring congener *M. sexta*) have a higher thermal tolerance than the shared parasitoid *C. congregata*, resulting in a thermal mismatch, (2) whether parasitized hosts of either species can partially recover CcBV-disrupted developmental pathways under high temperatures, and (3) whether host species differ in their response to parasitism and temperature. The results of this study indicate that *M. quinquemaculata* parasitized by *C. congregata* display a thermal mismatch under high temperatures similar to that seen in *M. sexta* (Fig. 1). Our findings also highlight important differences between these two sympatric congeners in their responses to parasitism and heat shock.

In this host–parasitoid system, heat shock or heat waves soon after parasitism (e.g. within the first 5 days after oviposition) result in complete parasitoid mortality and disruption of the parasitoid's manipulation of host development (Moore et al., 2021, 2022): final instar *M. sexta* larvae continue to feed and grow but fail to wander, molt or pupate following parasitism and heat shock. This host phenotype has also been experimentally generated by injection of CcBV virions (without parasitoid eggs), suggesting that the endosymbiotic virus plays an important role in host endocrine disruption to accommodate parasitoid development (discussed below) (Beckage et al., 1994). The present study shows that for *Manduca* from wild field-collected populations, heat shocks after parasitism allow some caterpillars to escape developmental disruption and successfully transition to the prepupal wandering stage. The magnitude of this effect was specific to the host species. For example, recovery of the wandering stage was much more common in *M. quinquemaculata* (47%) than in *M. sexta* (13%) (Fig. 1). These species-level differences are supported statistically: both the multinomial logistic regression (Table 1) and GLMs indicate that inclusion of species identity as a predictor term improved the models significantly. Under control temperatures, the parasitoid typically disrupts host development just prior to the wandering stage. Although high temperatures can facilitate host recovery of this developmental stage, hosts only very rarely progressed to pupation and adulthood. Only *M. quinquemaculata* displayed an instance of full developmental recovery of the adult stage under parasitized, heat-shocked conditions. High temperatures may therefore provide hosts a route of escape from the characteristically lethal parasitism by *C. congregata* in a host-species-specific manner*,* but only in extremely rare instances*,* and whether this phenomenon can result in fertile adults has not been demonstrated here.

The mechanisms underlying host differences in developmental recovery of the wandering stage are unknown, but stress responses likely play a role. Notably, *M. sexta* from a laboratory colony – which has not been exposed to heat stress or parasitoids for over 250 generations – do not show evidence for heat-induced recovery of the wandering stage after parasitism (Moore et al., 2021, 2022). Domesticated laboratory *M. sexta* have lower heat tolerance and reduced immune responses to immune challenges relative to field *M. sexta* potentially owing to relaxed selection for immune and thermal responses in the laboratory environment (Kingsolver and Nagle, 2007; Diamond and Kingsolver, 2010; Alston et al., 2020), a pattern seen in other laboratory and domesticated species (Lahti et al., 2009). Greater heat tolerance and immune function may therefore be associated with the heat-induced recovery of the wandering stage seen in field, but not laboratory, *M. sexta.* Consistent with this idea, *M. quinquemaculata* may be more tolerant of high temperatures than *M. sexta* with body size as a proxy for fitness: for unparasitized caterpillars, heat decreased mean final size (mass at wandering) in *M. sexta*, but increased final size in *M. quinquemaculata* (Fig. 3). Differences in thermal tolerance and immunity between species are likely important drivers of the ecological outcomes of species interactions in the face of heat waves and temperature extremes.

Similarly, developmental plasticity may be an important component of host species differences in the heat-induced recovery of the wandering stage: a key function of the parasitoid's endosymbiotic bracovirus is disruption of host endocrine pathways in the terminal instar to prevent host pupation (Beckage and Riddiford, 1983; Dushay and Beckage, 1993). Development of extranumery instars is an important response to stress in *Manduca*, Lepidoptera and other insects (Abarca et al., 2020; Albrecht, 1957; Hassall and Grayson, 1987). Field *M. sexta* have a greater propensity to develop extranumery instars than laboratory *M. sexta* under stressful conditions (Kingsolver and Nagle, 2007), and the results of our study suggest that in field *Manduca*, developmental flexibility may be greater in *M. quinquemaculata* than in *M. sexta:* in the double-treatment group, *M. quinquemaculata* developed extranumery instars at a higher frequency (69.3%) than *M. sexta* (29.6%) (Fig. S1). The ability to develop extranumery instars under stress parallels the ability of the host species or population to partially recover from parasitism following heat shocks, similar to the pattern of heat tolerance (and potentially immunity, but this has not been tested in *M. quinquemaculata*). This suggests that differences in developmental plasticity may play a role in a host's response to parasitism and heat stress. However, more work is needed to confirm a correlation between extranumery instar development and partial recovery from parasitism: sample sizes of *M. sexta* recovering the wandering stage of development were too low to detect significant differences between species exclusively for this developmental outcome (Fig. S2).

The proximal cause of the breakdown in typical ecological interactions between *C. congregata* and its hosts is unclear, but several studies may provide clues. First, a 2018 preliminary study subjected newly parasitized field *M. sexta* to temperatures ranging from warm to extremely hot (35, 40, 42, 43 and 44°C) for 24 h, then quantified the resulting host developmental outcomes. The results suggest there is a dosage-dependent effect of heat on development of the wandering phenotype, which indicates that the partial developmental recovery of parasitized hosts is temperature-dependent (details of this study can be found in Fig. S3). Second, *C. congregata*, like other parasitoid wasps, relies on its viral endosymbiont for successful parasitism and reproduction (Beckage et al., 1994; Beckage and Templeton, 1986), and a recent study in another host–parasitoid–viral endosymbiont system suggests thermal disruption of the viral endosymbiont's manipulation of the host may be a key factor in the thermal breakdown of ecological interactions between host and parasitoid. In this spruce budworm–ichneumonid wasp–*Ichnovirus* system, high temperatures reduce parasitoid survival, decrease ichnoviral gene expression and increase immune gene expression in the host (Seehausen et al., 2017). The effect of high temperatures on host immunosuppression by CcBV and its interaction with the host species/population is currently being studied in the *Manduca–C*. *congregata*–CcBV host–parasitoid–endosymbiont system.

Heat-induced disruptions in closely coupled species interactions, such as hosts and their parasitoids or pathogens, are not uncommon (Palmer-Young et al., 2018; Wernegreen, 2012). Parasites, pathogens and symbionts often have a lower thermal tolerance than their hosts, a key concept in behavioral or physiological fever, in which hosts raise their body temperatures to enhance immunity and/or decrease parasite or pathogen load (Adamo and McMillan, 2019). Beyond the potential host population-level benefits to decreasing the number of surviving parasitoids in subsequent generations, the results of the present study indicate some proximal therapeutic effect associated with high temperatures in parasitized caterpillars: parasitized hosts of both species had a higher probability of surviving to one of the other developmental outcomes (wasp emergence, WOWE or wandering) at 40°C than at 25°C (Fig. 2). *Manduca* are thermal generalists able to survive temperatures of 40°C for prolonged time periods, but heat stress compounded with other stressors typically reduces survival (Jørgensen et al., 2022). As the 40°C group experienced the added stressor of extreme high temperature exposure, this increased probability of survival suggests some immediate benefit to experiencing high temperatures in *Manduca* hosts. Many insects, including Lepidoptera, engage in behavioral fever in response to parasites, pathogens and parasitoids (Adamo and McMillan, 2019; Karban, 1998). Despite these potential benefits, recent studies show no evidence for behavioral fever in laboratory or field *M. sexta* parasitized by *C. congregata* (Johnston, 2020; Moore et al., 2023), and demonstrate that parasitized and unparasitized field *M. sexta* do not differ in body temperature throughout development on host plants in the field (Moore, 2021; Moore et al., 2022).

Parasitized hosts interact with a suite of other ecological players beyond the parasitoid and its viral endosymbiont in the field, and interactions with the host plant may also be ecologically relevant. Although hosts co-occur temporally and spatially, host species differ in their choice of feeding sites on tobacco host plants, which differ significantly in plant defensive chemical content. Within a leaf, *M. quinquemaculata* typically feeds on apical regions of tobacco leaves, which contain 2- to 3-fold higher nicotine content than the basal regions preferred by *M. sexta* (Kester et al., 2002). *Manduca* species, consistent with their feeding site preferences, differ in their nicotine tolerance – in electrophysiological assays, *M. quinquemaculata* had a higher chemosensory threshold for nicotine than *M. sexta* (Kester et al., 2002). Previous work demonstrated that increased nicotine concentrations can have immunotherapeutic effects in *M. sexta*, stimulating the melanization pathway important to parasitoid and parasite defense (Garvey et al., 2021), and reducing the survival of *C. congregata* larvae in parasitized hosts (Thurston and Fox, 1972; Thorpe and Barbosa, 1986). Nicotine content was held constant in the present study, but host plant allelochemicals can vary with temperature in the strength of their effects on herbivore biological processes, including immunity (Stamp and Osier, 1997; Diamond and Kingsolver, 2010). Whether host species differences in nicotine tolerance impact the response to parasitism, temperature and their interaction is unknown.

Despite the thermal mismatch between *C. congregata* and its hosts described in this and other studies (Moore et al., 2022, 2020, 2021), no evidence of WOWE phenotypes, nor host-species-specific differences in parasitism rates by *C. congregata*, have been found in the field thus far (Lawson, 1959) (K. Malinski and C. Sorenson, personal observations). We expect the former is likely because caterpillars of WOWE size and longevity do not last long in the presence of birds, wasps and other predators. The heat shock used in this study (40°C for 24 h) was chosen to capture low frequencies of larval developmental recovery from parasitism, but insects can differ in their responses to sudden novel high temperatures versus more ecologically relevant gradual temperature increases. A previous study using a more ecologically relevant heat wave (3 days at 30±10°C) demonstrated results consistent with those presented here: host–parasitoid thermal mismatch and similar rates of host developmental outcomes in parasitized *M. sexta* (Moore et al., 2022). By contrast, heat waves in the field vary in intensity, duration and frequency with respect to the timing of host and parasitoid development, and may generate a range of ecological outcomes, including wasp emergence, WOWEs and developmentally recovered wanderers. In order for thermal mismatch to occur in the field, adult wasps must oviposit into the host during or before a heat wave that is both long and hot enough to kill developing wasps. A 2019 study at our field sites demonstrated that temperatures experienced by caterpillars on their host plants reach highs above 40°C for several days during heat waves (Moore et al., 2022). The results of the present study suggest these temperatures can dramatically reduce wasp survival in both host species, which may reduce wasp population sizes. This may impact *Manduca* population dynamics indirectly via reduced parasitoid-related mortality under increased heat wave frequency and severity scenarios resulting from climate change. This is likely to be a more significant driver of host population dynamics than the small proportion of parasitized hosts that recover their wandering stage of development, as these hosts rarely survive to adulthood. Interestingly, parasitism rates at the same field sites in 2022 (one year after the present study) were extremely low (K. Malinski and C. Sorenson, personal observations). It is unclear whether the intense July 2022 heat wave is related, and whether these temperature extremes played a role in decreasing *C. congregata* survival in the field, but is worth noting here and following in future years.

The results of this study demonstrate the context-dependent nature of species interactions: biotic stressors such as parasites and parasitoids, and abiotic stressors such as environmental temperature can interact to reveal atypical ecological outcomes, even in closely related, co-occurring species. Extreme high temperatures altered the interaction between *Manduca* hosts and their *C. congregata* parasitoid, but to different magnitudes in the different host species. We caveat that this study was limited in its use of host and parasitoid populations, and the results of this study represent only one geographic location of many across the southeastern US. Whether the patterns demonstrated in this study are robust to these other *Manduca* and *C. congregata* populations across this range has not been investigated, but these results highlight the complexity in community-level responses to climate warming: substantial ecological shifts can occur even in species with no differences in susceptibility to a common parasite or parasitoid under normal temperature conditions. The downstream consequences of such effects in this and other host–parasitoid systems may be difficult to predict and depend on ecological context, but likely include reduced top-down control of hosts by parasitoids (Pardikes et al., 2022; Moore et al., 2020), and increased herbivory in urban and agricultural systems that rely on biocontrol of pests (Dale and Frank, 2014; Deutsch et al., 2018). Parasitoids are one of the most diverse groups of animals on the planet, and, as small-bodied ectotherms, are particularly vulnerable to the effects of climate change (Lister and Garcia, 2018; Barley et al., 2021; Wernegreen, 2012; Youngsteadt et al., 2015; Forbes et al., 2018; Colinet et al., 2015; Delava et al., 2016). We hope this study will provide a framework for increasing the ecological complexity of future investigations of climate change effects in other host–parasitoid systems. In the face of global insect population declines (Lister and Garcia, 2018; Hallmann et al., 2017; Hamblin et al., 2018), more work investigating the multitrophic impacts of climate warming in insect systems is particularly vital. Ultimately, we believe illuminating the responses of multitrophic systems to extreme high temperatures, as well as the mechanisms that govern them, will increase our understanding of community- and ecosystem-level responses to climate change.

## Supplementary Material

10.1242/jexbio.245702_sup1 Supplementary information
