# Supplementary material for: Host species differences in the thermal mismatch of host–parasitoid interactions
Source: J Exp Biol. 2023 Jun 27;226(12):jeb245702. doi: 10.1242/jeb.245702 (PMC10323234; doi:10.1242/jeb.245702)
Supplement: Supplementary information [file jexbio-226-245702-s1.pdf]

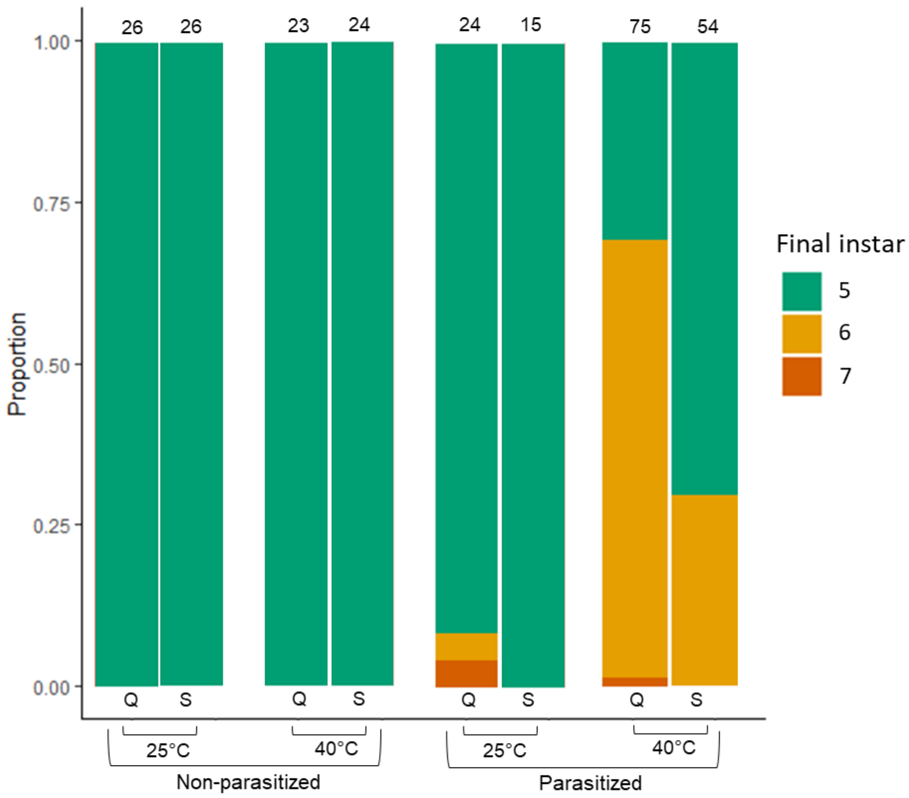

Fig. S1. Proportions of final instar number for hosts surviving to 5th instar by treatment groups. Treatment groups were: temperature (40°C, 25°C) and parasitism (P, parasitized, NP, not parasitized) for *M. sexta* (S) and *M. quinquemaculata* (Q) host species. Colors indicate final instar number: 5 (typical), 6 (extranumery), 7 (extranumery). Numbers above bars indicate sample sizes of respective treatment groups for individuals surviving to 5th instar.

Journal of Experimental Biology • Supplementary information

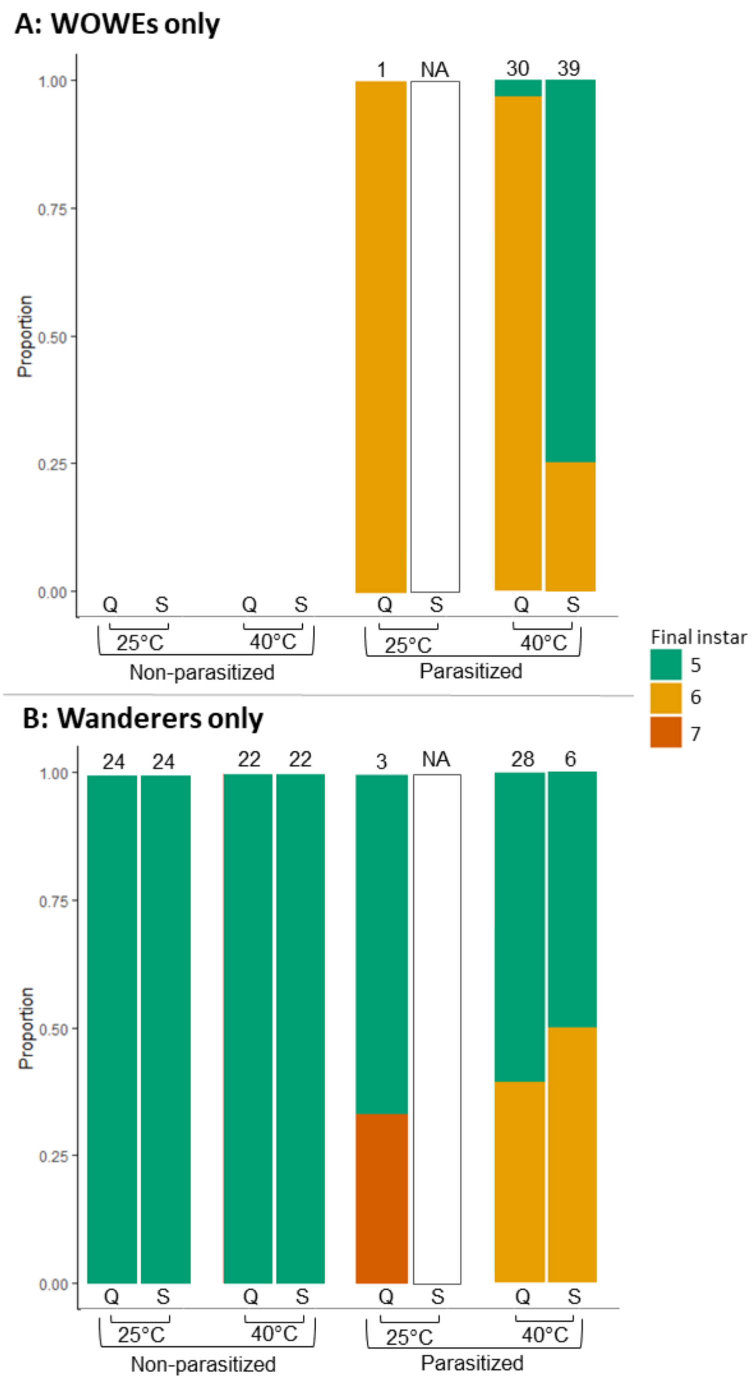

Fig. S2. Proportions of final instar number for hosts surviving to 5th instar by treatment groups, split by developmental outcome (WOWEs, panel A; wanderers, panel B). Treatment groups were: temperature (40°C, 25°C) and parasitism (P, parasitized, NP, not parasitized) for *M. sexta* (S) and *M. quinquemaculata* (Q) host species. No hosts with the outcome of wasp emergence developed extranumery instars. Blank bars indicate no hosts developing the respective outcome within the treatment group. Numbers above bars indicate sample sizes of respective treatment groups for individuals surviving to 5th instar with that particular developmental outcome.

Journal of Experimental Biology • Supplementary information

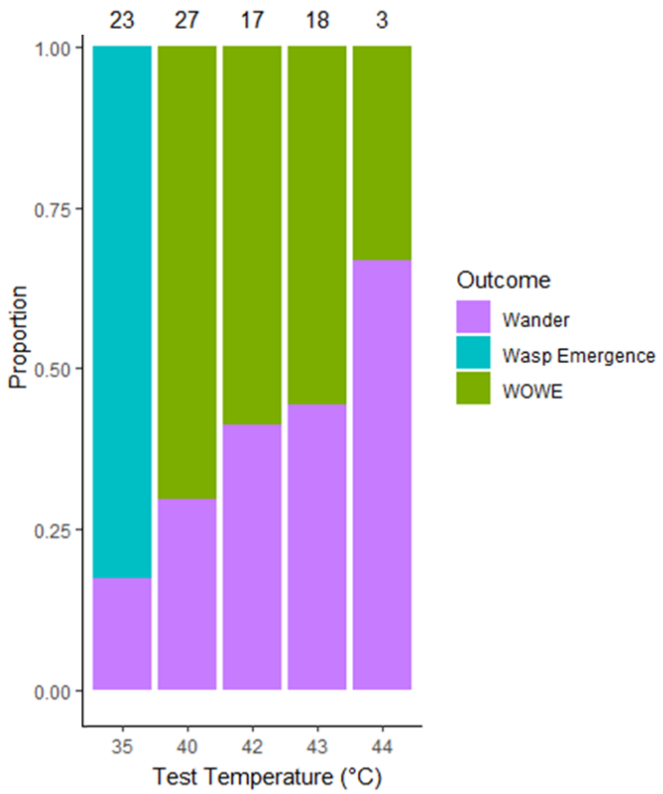

Fig. S3. Results of a study run in the summer of 2018 investigating the developmental outcomes of field *M. sexta* hosts parasitized by *C. congregata* and exposed to one of 5 high temperature treatments. Hosts were housed at 25ºC until 3rd instar; on the 1st day of 3rd instar, hosts in the parasitized treatment group were parasitized, and on the same day entered into a 24-hour exposure to the treatment temperature (35, 40, 42, 43, or 44ºC). Hosts were returned to 25ºC for the remainder of the experiment and followed until one of the following outcomes was observed: death (not shown), WOWE hosts (lime green), wasp emergence (turquoise), or wandering (purple). Non-parasitized control hosts displayed only the wandering outcome and are therefore not shown. Proportions of surviving hosts within treatment groups developing into each outcome are shown. Mortality by treatment group was: 50, 38, 34, 64, and 62% for non-parasitized, and 57, 41, 51, 75, and 93% for parasitized hosts at 35, 40, 42, 43, 44ºC, respectively. Parasitized wanderers were not tested for presence of CcBV, so failed ovipositions cannot be ruled out as a potential cause of different levels of wandering across treatments, but are highly unlikely because parasitized hosts were treated identically until after parasitism, and typical oviposition failure rates (parasitized wanderers developing at 25ºC, negative for CcBV via PCR assay) are ~10%.

Table S1. Results of the additive cox proportional hazards (coxph) model. Species, temperature, and parasitism were included as fixed variable terms. Coefficients (coef), standard errors of the coefficients (se(coef)), and p-values (Pr(>|z|) are shown. Reference levels were: *M. sexta* for species, 25°C for temperature, and not parasitized (NP) for parasitism. Significant values (p<0.05) are bolded, and marginally-significant values (p<0.1) are italicized.

Term	coef	se(coef)	Pr(>|z|)
Species	0.137	0.193	0.478
Temperature	-0.393	0.217	*0.071*
Parasitism	0.742	0.261	**0.004**

Journal of Experimental Biology • Supplementary information
